# Spectroscopic analysis reveals the effect of hairpin loop formation on G-quadruplex structures†

**DOI:** 10.1039/d2cb00045h

**Published:** 2022-03-10

**Authors:** Hengxin Feng, Chun Kit Kwok

**Affiliations:** Department of Chemistry and State Key Laboratory of Marine Pollution, City University of Hong Kong, Kowloon Tong Hong Kong SAR China ckkwok42@cityu.edu.hk +852 3442 0522 +852 3442 6858; Shenzhen Research Institute of City University of Hong Kong Shenzhen China

## Abstract

We study and uncover the effect of hairpin structures in loops of G-quadruplexes using spectroscopic methods. Notably, we show that the sequence, structure, and position of the hairpin loop control the spectroscopic properties of long loop G-quadruplexes, and highlight that intrinsic fluorescence can be used to monitor the formation of non-canonical G-quadruplexes.

## Abstract 2

This work studies the intrinsic fluorescence properties of long-loop G-quadruplexes (G4) with hairpin loop structures, revealing the unique information of G4 provided by intrinsic fluorescence compared to other spectroscopic assays.

## Graphical abstract

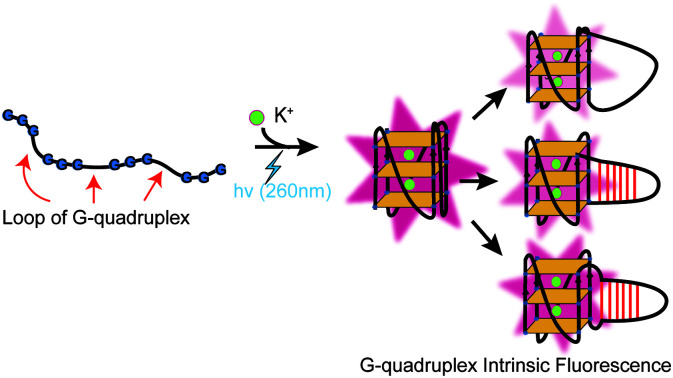

Guanine-rich (G-rich) regions in DNA and RNA can self-assemble into a G-quartet (Fig. 1A) through Hoogsteen base pairing.^1,2^ When stabilized by a monovalent cation such as a potassium ion (K^+^), G-quartets can stack to form a secondary structure referred to as a G-quadruplex (G4).^1,2^ A canonical G4 consists of three or more layers of G-quartet linked by a loop with 1–7 nucleotides (G_3+_N_1–7_G_3+_N_1–7_G_3+_N_1–7_G_3+_)^3^ (Fig. 1B). Canonical G4s in both DNA (dG4s) and RNA (rG4s) have been reported, and these G4s can interact with proteins to control gene functions.^2,4^ Currently, most G4 studies focused on canonical G4s; however, non-canonical G4s, such as two layers of quartet, bulged G4s, or G4s with a long loop, were recently reported in both the human genome and transcriptome.^5,6^ In addition, G4s with long loops capable of forming a hairpin structure have been discovered in HIV-1 genomic RNA^7^ and in the human genome.^8–10^ The latest studies of this emerging non-canonical G4 structure subtype reveal that the hairpin structure in the G4 loop can accelerate G4 folding,^11^ stabilize G4 and regulate gene expression.^8–10^ The importance of G4 with hairpin loops is underestimated and underexplored due to the conventional definition of G4 and related G4 prediction programs.^1,3^

**Fig. 1:**
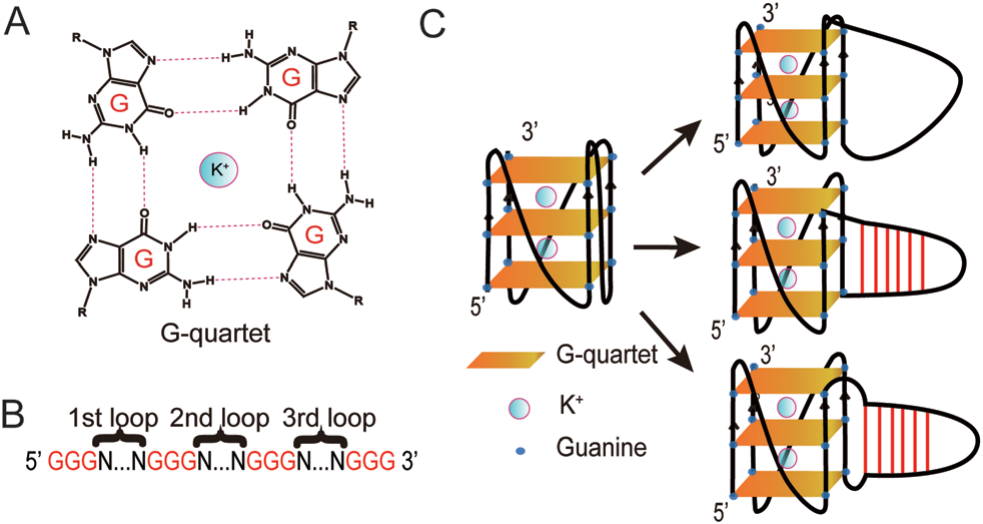
Schematic of the G-quartet and G4. (A) G-quartet chemical structure. The K^+^ stabilizes the G-quartet. (B) Schematic sequence of G4s studied. Gs in red are the guanines in the G-quartets. Ns between two sets of Gs represent the nucleotides in the first, second and third loops. (C) Left, structure of a G4 with a short loop. Right top, folding of a G4 with a long linear loop. Right middle, folding of a G4 with a hairpin loop. Right bottom, folding of a G4 with non-pairing Ts between the core of the G-quartet and the hairpin loop.

For decades, techniques including ultra-violet (UV) melting,^12^ circular dichroism (CD) titration,^13^ nuclear magnetic resonance (NMR),^14^ and fluorescence resonance energy transfer (FRET) melting^15^ have been used to study the spectroscopic signatures of G4s. In addition to these techniques, the intrinsic fluorescence of G4s was reported in DNA G4s^16^ and in RNA G4s.^17^ Factors affecting the intrinsic fluorescence have then been studied by several research groups including our laboratory.^18–24^ For example, the loop sequence and length have been reported to affect the G4 intrinsic fluorescence and dG3T possessed the highest intrinsic fluorescence intensity among those tested.^19^ Based on a dG3T scaffold, the effects of dangling nucleotides,^21^ cosolutes^21^ and bulges^23^ on G4 intrinsic fluorescence have also been studied. In addition, Curtis and colleagues reported that mutated G-quartets also exhibited intrinsic fluorescence and the intensity depends on the mutated nucleotide combinations.^22^ Although the effect of loop sequence on intrinsic fluorescence has been studied, the loop length used in those studies was short and limited to 3 nucleotides (nts).^18–24^ As such, little is known about the effect of long loops and loop base pairing on G4 spectroscopic features, particularly regarding their impacts on G4 intrinsic fluorescence.

In this study, besides dG3T, we designed a total of 34 dG4 constructs with different long loops to study their spectroscopic features, including CD, UV melting, and intrinsic fluorescence. We determined the thermostability and parallel topology of these constructs, and thereafter measured their intrinsic fluorescence. We revealed that the enhancement of the G4 intrinsic fluorescence is affected by the formation of a hairpin structure in the G4 loop. We further reported the effect of loop position, base pair number, loop sequence and non-pairing nucleotides between the G4 core and G4 loop on the intrinsic fluorescence of G4s.

Based on the model oligonucleotide dG3T used in our previous studies,^19,21,23^ we initially designed long loop dG4 constructs with a 15 nt long loop in either the 1st, 2nd or 3rd loop region of the G4, while other loop regions remained a single T nucleotide (Fig. 1B). Within the 15 nt in each loop region, the sequences are designed to contain either linear T loops (5′ T15 3′), or 2-base pair (5′ A2T13 3′), 4-base pair (5′ A4T11 3′), or 6-base pair (5′ A6T9 3′) loops (Fig. 1C). Long loop positions were labelled as 1st, 2nd and 3rd, as shown in Fig. 1B, and the number after denotes the number of relative nucleotides in the loop from 5′ to 3′, *e.g.* 1st A6T9 denoted the sequence GGGAAAAAATTTTTTTTTGGGTGGGTGGG.

We first studied the 12 constructs to test whether AT base pairing could affect their intrinsic fluorescence and G4 formation under 150 mM K^+^ conditions. We observed that the intensity of intrinsic fluorescence increased from T15 to A6T9 in both the 2nd and 3rd loop, whereas the intensities of intrinsic fluorescence of oligos with the 1st loop were similar (Fig. S1, ESI†). All G4s with long loops had lower intrinsic fluorescence than dG3T (Fig. S1, ESI†). To examine the topology of the G4s, we performed CD and observed a positive peak at 263 nm and a negative peak at around 240 nm in the spectra (Fig. S2, ESI†), suggesting the formation of parallel G4 topology. To assess the thermostability of G4, we conducted UV melting and found the hypochromic shift in UV melting monitored at 295 nm, with the melting temperatures (*T*_m_) all above 37 °C, indicating that the G4s are thermostable (Fig. S3, ESI†). As dG3T is very stable under 150 mM K^+^, the *T*_m_ cannot be accurately obtained (>90 °C) (Fig. S3, ESI†). Therefore, we also performed the same reactions using 15 mM K^+^ conditions. We found that the patterns of CD spectra for these G4 oligos under 15 mM and 150 mM K^+^ were consistent and suggestive of parallel topology (Fig. S4, ESI†). The quantum yield of dG3T in 15 mM K^+^ buffer was determined to be 2.3 × 10^−3^, which is consistent with what was previously reported.^21^ The result of UV melting showed that the *T*_m_s of the dG4s were ranging from 44 to 51.25 °C (Fig. S5 and Table S2, ESI†), with 
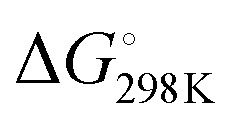
 ranging from −29.5 to −15.8 kJ mol^−1^, and 
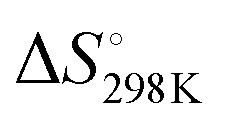
 ranging from −1211.4 to −584.4 J K^−1^ mol^−1^ (Table S2, ESI†). The *T*_m_ of dG3T under 15 mM K^+^ was 89 °C (Fig. S5, ESI†), with 
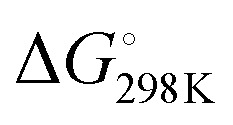
 and 
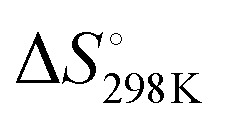
 of −104.2 kJ mol^−1^ and −1724.8 J K^−1^ mol^−1^, respectively (Table S2, ESI†). Despite the lowering in K^+^, the trend of intrinsic fluorescence for the 12 constructs remains similar (Fig. 2 and Table S1, ESI†). The spectra were normalized with the standard of dG3T emission at 386 nm, *i.e.* the emission maxima peak of dG3T. Notably, we observed that the intrinsic fluorescence at 386 nm increased with increasing base pair number in the hairpin loop, *i.e.* from T15 to A6T9, in all three loops (Fig. 2A). We found that 2nd A4T11 and A6T9 showed the highest intensity of intrinsic fluorescence in the tested oligos (Fig. 2A), suggesting that the intrinsic fluorescence of dG4 is not only affected by loop length but also the base pairing in the hairpin loop. The oligos did not show G4 features in intrinsic fluorescence nor CD spectra under 15 mM Li^+^ conditions (Fig. S6, ESI†), verifying that these spectroscopic features are caused by the structure and not the sequence. Overall, our data supported the formation of parallel, thermostable long loop G4s, and reported for the first time that they can exhibit intrinsic fluorescence, and their intensities can be affected by base pairing within the loop region.

**Fig. 2:**
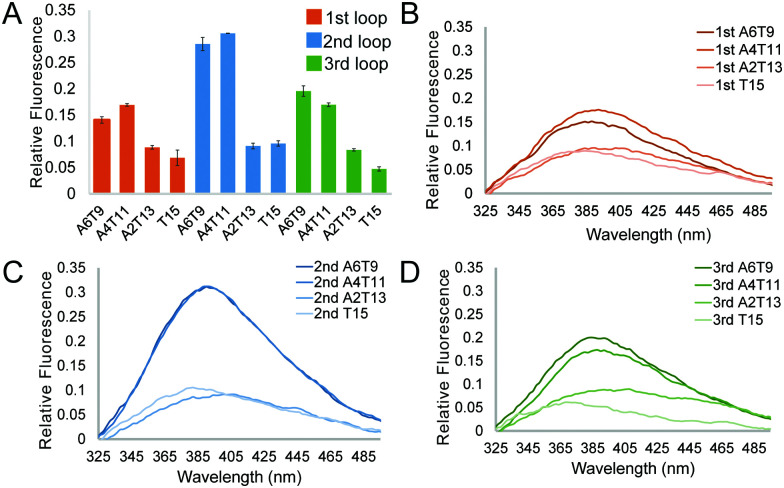
Effect of the number of base pairs in a loop and the loop position on the G4 intrinsic fluorescence. (A) Relative fluorescence intensity of G4s with a long loop in different positions and numbers of base pairs in 15 mM K^+^. Data show the average of three replicates and the error bars represent the standard errors. (B–D) are the representative plots of loops with different numbers of base pairs in the 1st, 2nd and 3rd loop, respectively.

To further investigate the effect of loop base pairing on dG4, we next designed 9 constructs with the loops of T13A2, T11A4, and T9A6 in different loop positions (Table S1, ESI†). These dG4 constructs contain hairpin loops with the same number of base pairs, with AT to TA base pair flip in the sequence and secondary structure design (Table S1, ESI†). We tested their intrinsic fluorescence spectra (Fig. S7, ESI†) and conducted CD and UV melting to determine the thermostability of dG4 and the parallel topology of dG4s in these 9 new constructs (Fig. S8 and S9, ESI†). Similar to the results before (Fig. 2), the intrinsic fluorescence of these new constructs decreased with decreasing number of base pairs, with the only exception that 2nd T13A2 had somewhat lower intensity than 2nd T15, respectively (Fig. 3A and Table S2, ESI†). Compared to dG4s with the A6T9 loops in all three loop positions, dG4s with T9A6 loops showed higher intrinsic fluorescence intensities, while T11A4 and T13A2 loops had lower intensities than A4T11 and A2T13 loops (Fig. 3B and Table S2, ESI†), suggesting that the AT and TA base pairings in each loop contribute differently to the intrinsic fluorescence intensity.

**Fig. 3:**
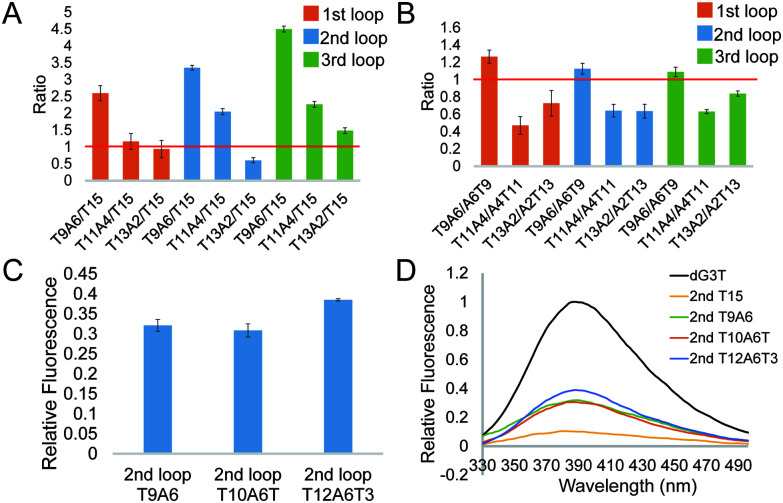
Effect of the T–A base position in the loop and the non-pairing Ts between the G cores and the loop on the intrinsic fluorescence intensity of the G4. (A) Comparison between G4s with base pairs in a hairpin loop and a linear long loop, in which the Ts in the base pairs are closer to the 5′ end. (B) Change in fold between the G4s with the same base pairing number and different A–T positions. (C) Relative fluorescence of 2nd T9A6, T10A6T and T12A6T3. Average of three replicates are shown and error bars represent the standard errors. (D) Representative intrinsic fluorescence plots of G4s with different 2nd loops.

The constructs tested above all had the same TTT trinucleotide loop, *i.e.* the non-pairing part of the hairpin structure. To test if different trinucleotide loops may influence the intrinsic fluorescence intensity of dG4s with hairpin loops, we designed constructs with AAA trinucleotide loops (5′ T6A9 3′) in different loop regions (Table S1, ESI†). A previous study reported that GNA trinucleotides can stabilize DNA hairpin loops.^25^ We designed constructs with 6 TA base pairs and GAA or GCA trinucleotide loops, *i.e.* 5′ T6GA8 3′ and 5′ T6GCA7 3′ (Table S1, ESI†). The intrinsic fluorescence intensity of 1st T6A9 was higher than that of 1st T9A6, whereas other constructs with a T6A9 loop showed lower intrinsic fluorescence intensity than the relative T9A6 constructs (Table S2, ESI†). In addition, 3rd T6GCA7 showed higher intrinsic fluorescence intensity than 3rd T6A9, while other constructs with GAA or GCA trinucleotide loops all showed intrinsic fluorescence intensity lower than the relative T6A9 constructs (Fig. S10 and Table S2, ESI†). The formation of dG4 and the parallel topology of the 9 new constructs were confirmed by CD and UV melting (Fig. S11 and S12, ESI†). Generally, dG4s with AAA, GAA or GCA trinucleotide loops had lower intrinsic fluorescence intensity compared with the relative T9A6 constructs, *i.e.* TTT trinucleotide loops.

From the results above we found that 2nd T9A6 had the highest relative intrinsic fluorescence intensity, 0.321 ± 0.014, which is more than 6 folds higher than the lowest one, 3rd T15 (0.047 ± 0.004). To assess whether switching some of the base pairs in the hairpin loop will change the intrinsic fluorescence intensity, we designed 2nd A3T6A3T3 and T3A3T6A3 (Table S1, ESI†). These two new constructs both had six base pairs, in which the positions of TA in three base pairs, either the three pairs closer to the G4 core or the ones closer to the trinucleotide loop, were switched to AT base pairs. The intrinsic fluorescence intensities of the two new constructs were both lower than 2nd T9A6, and 2nd T3A3T6A3 had lower intensity than 2nd A3T6A3T3 (Fig. S13A and Table S1, ESI†). The parallel topologies and thermostability of the new constructs were confirmed by CD and UV melting, respectively (Fig. S13B, C and Table S2, ESI†). Thus, changing the TA positions did not further increase the intrinsic fluorescence intensity based on 2nd T9A6. We also evaluated whether non-base pairing nucleotides between the base pairing stem and the G4 core (Fig. 1C right bottom), which were used in the kinetic study of long loop G4s,^11^ would increase the G4 intrinsic fluorescence intensity. To do so, we designed two new constructs with one or three pairs of non-pairing Ts, *i.e.* 2nd T10A6T and 2nd T12A6T3, respectively (Table S1, ESI†). The parallel topologies and stability at room temperature were confirmed by CD and UV melting, respectively (Fig. S14 and Table S2, ESI†). 2nd T10A6T did not change the intrinsic fluorescence intensity much, whereas 2nd T12A6T3 showed a significant increase in intensity compared to 2nd T9A6 (Fig. 3C). Overall, from the representative spectra (Fig. 3D) we observed that in the second loop position, intra-loop base pairing and non-pairing Ts enhanced the intrinsic fluorescence intensity compared to the ones with linear long loops. The long-loop G4 with the highest intrinsic fluorescence intensity was 2nd T12A6T3 in the constructs tested in this study, with relative intensity of 0.385 ± 0.003, which is about 8 fold higher than the lowest one. Oligos under 15 mM Li^+^ (Fig. S15, ESI†) and scrambled oligos, with the same nucleotide composition but unable to form G4 structures, under 15 mM K^+^ (Table S1 and Fig. S16, ESI†) showed no G4 signals in intrinsic fluorescence and CD assays, further supporting that the spectroscopic features were caused by the G4 structure.

We further studied the overall effect of base pair number in the G4 loop on the intrinsic fluorescence intensity. We grouped the relative intrinsic fluorescence intensity and CD peak wavelength data according to the base pair number in the loops and generated box plots for each group (Fig. 4A). The data showed a trend that the relative intrinsic fluorescence intensity increased with increasing number of base pairs in the loop, regardless of the long loop position, while the peaks of the CD spectra showed negative correlation with the hairpin loop base pair number. Correlation analysis between the intrinsic fluorescence and peak wavelength in CD showed a strong inverse correlation with *R* = −0.77 (Fig. 4B). Since dG3T has the relative intrinsic fluorescence of 1 and CD peak wavelength of 263 nm, hairpin loop formation leads to less alteration of G4 spectroscopic features in long loop G4s.

**Fig. 4:**
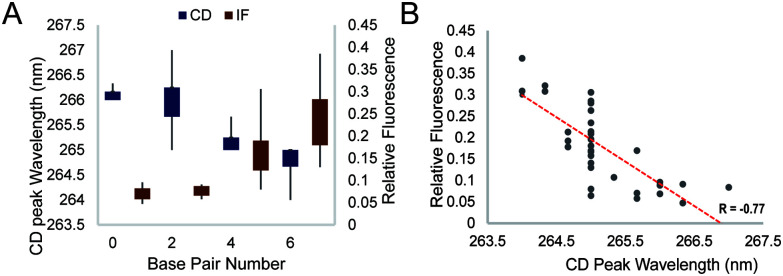
General effect of base pair number on the CD peak wavelength and intrinsic fluorescence. (A) Effect of base pair number on the CD peak wavelength and the relative fluorescence at 386 nm. (B) Correlation between the relative fluorescence at 386 nm and the wavelength of the peak in the CD spectra. Data points are generated from the average of three replicates. An inverse correlation is shown with *R* = −0.77.

Integrating the results from this work and our previous studies,^19,21,23^ we found that the dG3T construct remained the highest intrinsic fluorescence G4 that we have reported so far. In this study, we demonstrated that longer loops in G4 negatively impact the intrinsic fluorescence when compared to dG3T. We also observed that with the same loop sequence, long loops in the 2nd loop generally show a higher intrinsic fluorescence intensity than those in other loop positions (Table S1, ESI†). Interestingly, we found that loop length is not the only factor affecting the intrinsic fluorescence property. With the same loop length, intrinsic fluorescence is positively correlated to the base pair number (Fig. 4A), and can be influenced by the orientation of the base pairs and the sequence context of the loop as well (Fig. 3B and Fig. S10, ESI†). Moreover, we showed that the non-base pairing Ts at the G4 – intra-loop base pair junction can lead to enhancement in G4 intrinsic fluorescence (Fig. 3C).

Previous reports have suggested that increasing the number of base pairs can enhance the stability of the hairpin structure^26^ and the stability of hairpins with different AT and TA combinations is different.^27^ Therefore, it is reasonable for us to observe the effect of base pair number and loop sequence in the hairpins on G4 intrinsic fluorescence. Interestingly, while we and recently others,^9^ found that the stability of G4s with the same long loop sequence in different G4 loop positions was similar (Table S2, ESI†), here we showed that they exhibited a big difference in intrinsic fluorescence (Fig. 2A and Table S1, ESI†), suggesting that thermostability alone cannot explain the variation of G4 intrinsic fluorescence observed. This finding is consistent with our previous study on bulged G4 as well.^23^ It is of note that all G4s studied in this work were in parallel topology. These data indicated that besides thermostability and topology, other factors have roles in the intrinsic fluorescence of G4s, showing that intrinsic fluorescence can provide additional information on the G4 structure. We found that formation of a hairpin structure in the G4 loop position reduced the alteration in the CD feature (G4 core topology) and enhanced the intrinsic fluorescence compared to a linear long loop (Fig. 4A). We reasoned that the loop internal base pairing likely rigidifies the G4 core and/or enhances the stacking of the G4 planes, which leads to improvement in intrinsic fluorescence. This work, as part of the systematic study in our group,^19,21,23^ provides new and important findings of intrinsic fluorescence of non-canonical dG4s with long loops. Moreover, the intrinsic fluorescence of G4s is different when compared to other DNA or RNA structures.^28^ Up to now, intrinsic fluorescence has been applied in different G4 studies,^18–24^ which can be further explored for label-free and high-throughput G4 detection that can supplement other G4 detection techniques.

In sum, we have investigated the spectroscopic properties of long loop G4s using CD titration, UV melting and intrinsic fluorescence, and uncovered the effect of hairpin loop formation on this non-canonical G4 structure subtype. Notably, we have shown that intrinsic fluorescence can be used to detect the formation of unimolecular, three-plane, parallel long loop G4s for the first time, and identified key parameters that influenced the fluorescence properties between long loop G4s with or without a hairpin structure. This can also be easily applied to any G4 systems provided that both Li^+^ and K^+^ conditions are performed for comparison and G4 detection. The method reported here is label-free and involves no exogenous fluorophore, and can be used as a general fluorescence-based assay to detect canonical and non-canonical G4 formation.

## Conflicts of interest

There are no conflicts to declare.

## Supplementary Material

CB-003-D2CB00045H-s001
