# Supplementary material for: Spectroscopic analysis reveals the effect of hairpin loop formation on G-quadruplex structures
Source: RSC Chem Biol. 2022 Mar 10;3(4):431–5. doi: 10.1039/d2cb00045h (PMC8984947; doi:10.1039/d2cb00045h)
Supplement: CB-003-D2CB00045H-s001 [file CB-003-D2CB00045H-s001.pdf]

This journal is © The Royal Society of Chemistry 2022

Supporting Information

Methods
Table S1 Oligo sequences used in this study and their abbreviations, relative fluorescence, and CD peak wavelength.
Table S2 Thermodynamic data of oligos used in this study.
Fig. S1 Intrinsic fluorescence plot of A6T9, A4T11, A2T13 as well as dG3T in different loop positions in 150 mM K^+.
Fig. S2 CD spectra of A6T9, A4T11, A2T13 as well as dG3T in different loop positions in 150 mM K^+.
Fig. S3 UV melting spectra of A6T9, A4T11, A2T13 as well as dG3T in different loop positions in 150 mM K^+.
Fig. S4 CD spectra of A6T9, A4T11, A2T13 as well as dG3T in different loop positions in 15 mM K^+.
Fig. S5 UV melting spectra of A6T9, A4T11, A2T13 as well as dG3T in different loop positions in 15 mM K^+.
Fig. S6 Relative intrinsic fluorescence plots of T9A6, T11A4 and T13A2 in different loop positions in 15 mM K^+.
Fig. S7 CD spectra of T9A6, T11A4 and T13A2 in different loop positions in 15 mM K^+.
Fig. S8 UV melting spectra of T9A6, T11A4 and T13A2 in different loop positions in 15 mM K^+.
Fig. S9 Relative intrinsic fluorescence plots of T6A9, T6GA8 and T6GCA7 in different loop positions in 15 mM K^+.
Fig. S10 CD spectra of T6A9, T6GA8 and T6GCA7 in different loop positions in 15 mM K^+.
Fig. S11 UV melting spectra of T6A9, T6GA8 and T6GCA7 in different loop positions in 15 mM K^+.
Fig. S12 Relative intrinsic fluorescence plots, CD and UV melting spectra of 2nd A3T6A3T3 and 2nd T3A3T6A3 in 15 mM K^+.
Fig. S13 CD and UV melting spectra of 2nd T10A6T and 2nd T12A6T3 in 15 mM K^+.
References

Methods

DNA Preparation

In this study, DNA oligonucleotides were commercially supplied by Integrated DNA Technologies (IDT). Sequences, as well as abbreviations are shown in Table S1. The oligonucleotides were dissolved to the concentration of 100 µM with ultra-pure nuclease-free water (Invitrogen). Nano-Drop 1000 spectrophotometer (Thermo Scientific) was used to confirm the concentration of DNA oligonucleotides. Oligonucleotides were stored at -20°C before experiment.

Circular Dichroism (CD)

In this study, Jasco J-1500 CD spectrophotometer and Quartz cuvettes with the path length of 1 cm (Hellma Analytics) were used to perform CD spectroscopy. Samples were prepared in a reaction volume of 2 mL in total, containing DNA oligonucleotides with the final concentration of 5 µM, and a reaction salt and buffer of 15 mM KCl or LiCl, and 10 mM LiCac (pH 7.0). Samples were heated at 95 °C in Thermo-Shaker (ALLSHENG) for 5 minutes for denaturation and, thereafter, renatured by cooling to room temperature for around 15 minutes. The samples were excited and scanned between 220 – 310 nm at room temperature and spectra were obtained every 1 nm. Accumulation of 2 scans with a 2 s/nm response time were collected and averaged[1, 2]. The data were then normalized and then smoothed over 5 nm[3]. Data was processed and examined with Microsoft Excel.

Fluorescence Spectroscopy

HORIBA FluoroMax-4 and Quartz cuvettes with the path length of 1 cm (Hellma Analytics) were used to perform fluorescence spectroscopy in this study. Samples with a reaction volume of 2 mL in total were prepared, containing DNA oligonucleotides with final concentration of 5 µM, and a reaction salt and buffer of 15 mM KCl or LiCl, and 10 mM LiCac (pH 7.0). Samples were heated at 95 °C in Thermo-Shaker (ALLSHENG) for 5 minutes and then allowed to cool down at room temperature for around 15 minutes. The excitation wavelength of the G-quadruplex containing samples was set at 260 nm and then the emission spectra were collected between 300 – 500 nm as reported in previous study[1]. Spectra were obtained every 2 nm at room temperature. The bandwidth was set at 5 nm for both entrance and exit slits. For each oligonucleotide, the data was smoothed over 5 nm. Normalization of spectra was performed relative to the emission intensity of dG3T at 386 nm, i.e. the peak emission of dG3T. dG3T samples were analyzed under the same condition within the same day of the normalized samples. Three independent experiments were performed for each DNA oligonucleotide and results were analyzed with Microsoft Excel. Quantum yield of dG3T in 15 mM K^+ was obtained with the method previously reported by Sherlock et al[4].

Thermal Denaturation Monitored by UV Spectroscopy (UV Melting)

Samples containing 5 µM DNA oligonucleotides were prepared in a solution with 15 mM KCl or LiCl, and 10 mM LiCac (pH 7.0) in a reaction volume of 2 mL in total. All samples were denatured at 95 °C for 5 minutes, and cooled down to room temperature for around 15

minutes for renaturation. Agilent Cary 100 UV-Vis Spectrophotometer and Quartz cuvettes with the path length of 1 cm (Hellma Analytics) were used to conduct the UV melting experiments. After loading the sample solutions, the cuvettes were sealed with 3 layers of Teflon tape to prevent vaporization at high temperature. The samples were monitored at 295 nm from 20 to 95 °C with a temperature ramping rate of 0.5 ˚C/min. When temperature reached 95 °C, it was hold for 5 minutes. Following the holding time, a reversed scan at 295 nm was performed with a decreasing rate of 0.5 °C/min until 20 °C.

The initial data obtained were corrected by the blank solutions, which contains 15 mM KCl and 10 mM LiCac (pH 7.0) with a reaction volume of 2 mL in total. The data of each DNA oligonucleotide was then smoothed over 11 nm and Microsoft Excel was used to plot its first derivative. The final melting temperature was calculated by averaging the melting temperatures obtained from the forward and reverse scans.

For all the G4 samples, enthalpy (ΔH°) and entropy (ΔS°) were calculated based on the UV melting curve as described in previous research[5] following equations below, assuming change of folded and unfolded form in specific heat capacity (ΔCp°) ~ 0. In the equation, θ is the fraction of folded oligos and T is the relative temperature.

$$\text{Folding Constant } (K_a) = \frac{[\text{G4 folded}]}{[\text{Single strand DNA}]} = \frac{\theta(T)}{1 - \theta(T)}$$

$$\Delta G = -RT\ln(K_a) = \Delta H^o - T\Delta S^o$$

$$\ln(K_a) = \frac{-\Delta H^o}{RT} + \frac{\Delta S^o}{R}$$

Table S1 Oligo sequences used in this study and their abbreviations, relative fluorescence, and CD peak wavelength.

Long Loop Position	Oligo Abbreviation	Sequence	Relative Fluorescence	CD Peak Wavelength (nm)
-	dG3T	GGGTGGGTGGGTGGG	1	263.0
**1st loop**	1st T15	GGGTTTTTTTTTTTTTTTGGGTGGGTGGG	0.069 ± 0.015	266.0
	1st A6T9	GGGAAAAAATTTTTTTTTGGGTGGGTGGG	0.141 ± 0.006	265.0
	1st A4T11	GGGAAAATTTTTTTTTTTGGGTGGGTGGG	0.169 ± 0.002	265.0
	1st A2T13	GGGAATTTTTTTTTTTTTGGGTGGGTGGG	0.089 ± 0.004	266.0
	1st T9A6	GGGTTTTTTTTTAAAAAAGGGTGGGTGGG	0.178 ± 0.011	264.7
	1st T11A4	GGGTTTTTTTTTTTAAAAGGGTGGGTGGG	0.080 ± 0.008	265.0
	1st T13A2	GGGTTTTTTTTTTTTTAAGGGTGGGTGGG	0.064 ± 0.009	265.0
	1st T6GA8	GGGTTTTTTGAAAAAAAAGGGTGGGTGGG	0.130 ± 0.015	265.0
	1st T6GCA7	GGGTTTTTTGCAAAAAAAGGGTGGGTGGG	0.158 ± 0.005	265.0
	1st T6A9	GGGTTTTTTAAAAAAAAAGGGTGGGTGGG	0.209 ± 0.007	265.0
**2nd loop**	2nd T15	GGGTGGGTTTTTTTTTTTTTTTGGGTGGG	0.096 ± 0.005	266.0
	2nd A6T9	GGGTGGGAAAAAATTTTTTTTTGGGTGGG	0.286 ± 0.013	265.0
	2nd A4T11	GGGTGGGAAAATTTTTTTTTTTGGGTGGG	0.306 ± 0.001	265.0
	2nd A2T13	GGGTGGGAATTTTTTTTTTTTTGGGTGGG	0.091 ± 0.005	266.3
	2nd T9A6	GGGTGGGTTTTTTTTTAAAAAAGGGTGGG	0.321 ± 0.015	264.3
	2nd T11A4	GGGTGGGTTTTTTTTTTTAAAAGGGTGGG	0.196 ± 0.014	265.0
	2nd T13A2	GGGTGGGTTTTTTTTTTTTTAAGGGTGGG	0.058 ± 0.003	265.7
	2nd T6GA8	GGGTGGGTTTTTTGAAAAAAAAGGGTGGG	0.235 ± 0.006	265.0
	2nd T6GCA7	GGGTGGGTTTTTTGCAAAAAAAGGGTGGG	0.281 ± 0.009	265.0

	2nd T6A9	GGGTGGGTTTTTTAAAAAAAAAGGGTGGG	0.308 ± 0.015	264.3
	2nd A3T6A3T3	GGGTGGGAAATTTTTTAAATTTGGGTGGG	0.263 ± 0.011	265.0
	2nd T3A3T6A3	GGGTGGGTTTAAATTTTTTAAAGGGTGGG	0.167 ± 0.009	265.0
	2nd T10A6T	GGGTGGGTTTTTTTTTTAAAAAATGGGTGGG	0.309 ± 0.016	264.0
	2nd T12A6T3	GGGTGGGTTTTTTTTTTTTAAAAAATTTGGGTG<br>GG	0.385 ± 0.003	264.0
**3rd loop**	3rd T15	GGGTGGGTGGGTTTTTTTTTTTTTTTGGG	0.047 ± 0.004	266.3
	3rd A6T9	GGGTGGGTGGGAAAAAATTTTTTTTTGGG	0.196 ± 0.010	265.0
	3rd A4T11	GGGTGGGTGGGAAAATTTTTTTTTTTGGG	0.170 ± 0.004	265.7
	3rd A2T13	GGGTGGGTGGGAATTTTTTTTTTTTTGGG	0.084 ± 0.002	267.0
	3rd T9A6	GGGTGGGTGGGTTTTTTTTTAAAAAAGGG	0.213 ± 0.004	264.7
	3rd T11A4	GGGTGGGTGGGTTTTTTTTTTTAAAAGGG	0.107 ± 0.001	265.3
	3rd T13A2	GGGTGGGTGGGTTTTTTTTTTTTTAAGGG	0.070 ± 0.001	265.7
	3rd T6GA8	GGGTGGGTGGGTTTTTTGAAAAAAAAGGG	0.181 ± 0.010	265.0
	3rd T6GCA7	GGGTGGGTGGGTTTTTTGCAAAAAAAGGG	0.214 ± 0.005	265.0
	3rd T6A9	GGGTGGGTGGGTTTTTTAAAAAAAAAGGG	0.192 ± 0.009	264.7
**Scrambled Oligos**	Scr_T9A6	GGAAAGTGTTTGGTGTTTGGATTGAATGG	-	-
	Scr_T11A6	GGTTTGTAGTTGGTTAATGTTAGGTTGAAGG	-	-
	Scr_T15A6	GTTTGGTTTGGTTTGTTATTGTAAGGTGAATGT<br>AG	-	-

Note: Under the condition of 15 mM K^+.

Table S2 Thermodynamic data of oligos used in this study.

	Tm (°C)	ΔH (kJ mol^{-1})	$ΔS^0$ (J K^{-1} mol^{-1})	$ΔG^0_{298K}$	$ΔG^0_{310K}$
dG3T	89	-618.1	-1724.8	-104.2	-83.5
1st T15	47	-336.6	-1041.9	-26.1	-13.6
1st A6T9	49	-330.9	-1021.8	-26.4	-14.1
1st A4T11	46.25	-264.7	-825.3	-18.8	-8.9
1st A2T13	46.75	-246.7	-761.1	-19.9	-10.8
1st T9A6	48.25	-302.5	-938.4	-22.9	-11.6
1st T11A4	47	-213.1	-662.1	-15.8	-7.8
1st T13A2	47.5	-354.4	-1095.1	-28.1	-15.0
1st T6GA8	49	-299.2	-925.7	-23.3	-12.2
1st T6GCA7	50	-328.8	-1015.3	-26.3	-14.1
1st T6A9	46.75	-250.8	-782.7	-17.6	-8.2
2nd T15	46	-367.5	-1144.1	-26.6	-12.9
2nd A6T9	48.75	-331.3	-1027.9	-25.0	-12.7
2nd A4T11	46	-327.1	-1020.8	-22.9	-10.6
2nd A2T13	44.5	-368.0	-1146.1	-26.5	-12.7
2nd T9A6	46.5	-308.7	-965.0	-21.1	-9.5
2nd T11A4	44.75	-251.5	-787.7	-16.7	-7.3
2nd T13A2	46.5	-327.9	-1018.9	-24.3	-12.1
2nd T6GA8	47.75	-300.1	-933.5	-22.0	-10.8
2nd T6GCA7	49	-335.0	-1038.1	-25.7	-13.2
2nd T6A9	45	-255.2	-800.4	-16.7	-7.1
2nd A3T6A3T3	47.75	-314.8	-981.4	-22.3	-10.5
2nd T3A3T6A3	47.25	-295.7	-920.4	-21.4	-10.4
2nd T10A6T	47.5	-383.2	-1192.9	-27.7	-13.4
2nd T12A6T3	44.5	-388.0	-1211.4	-27.0	-12.4
3rd T15	48.75	-361.5	-1114.1	-29.5	-16.2
3rd A6T9	50	-280.5	-864.8	-22.8	-12.4
3rd A4T11	48.5	-228.1	-709.2	-16.8	-8.3
3rd A2T13	49.5	-338.8	-1043.0	-28.0	-15.5
3rd T9A6	48.5	-313.4	-976.5	-22.4	-10.7
3rd T11A4	48	-261.8	-812.1	-19.8	-10.1
3rd T13A2	49	-275.5	-853.8	-21.1	-10.9
3rd T6GA8	50	-330.8	-1022.6	-26.1	-13.8
3rd T6GCA7	51.25	-192.4	-584.4	-18.2	-11.2
3rd T6A9	48	-247.8	-770.5	-18.2	-8.9

Note: Under the condition of 15 mM K^+.

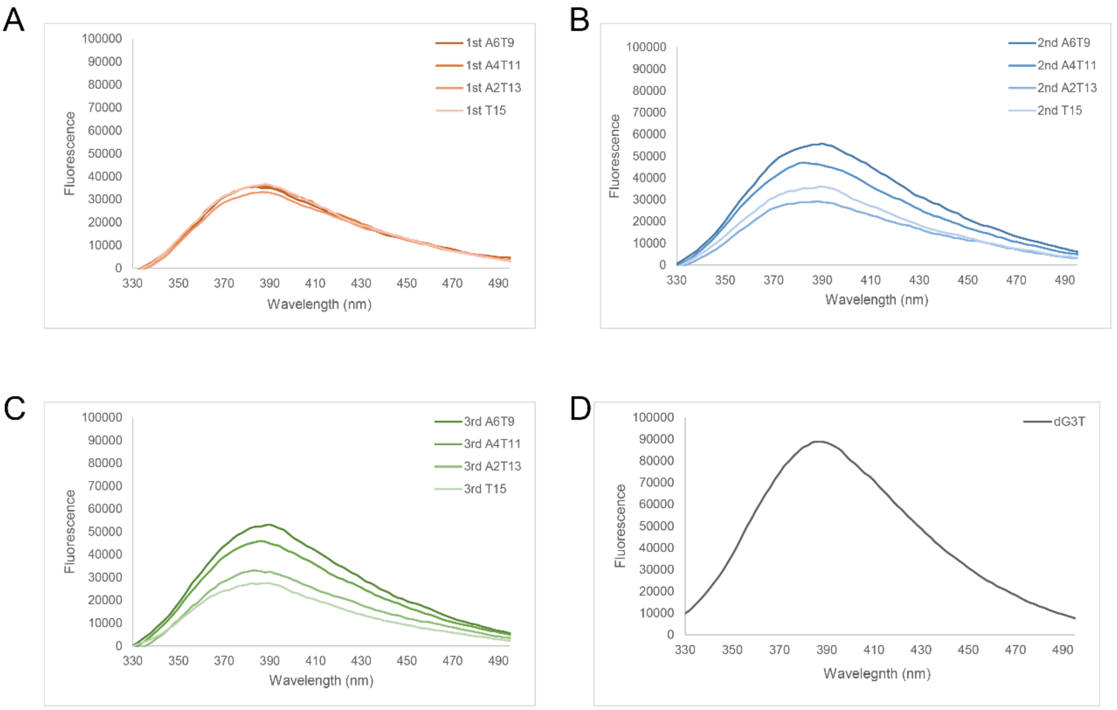

Fig. S1 Intrinsic fluorescence plot of A6T9, A4T11, A2T13 as well as dG3T in different loop positions in 150 mM K^+. (A), (B) and (C) are the representative plot of loops with different number of base pairs in first, second, and third loop respectively. (D) is the representative plot of dG3T.

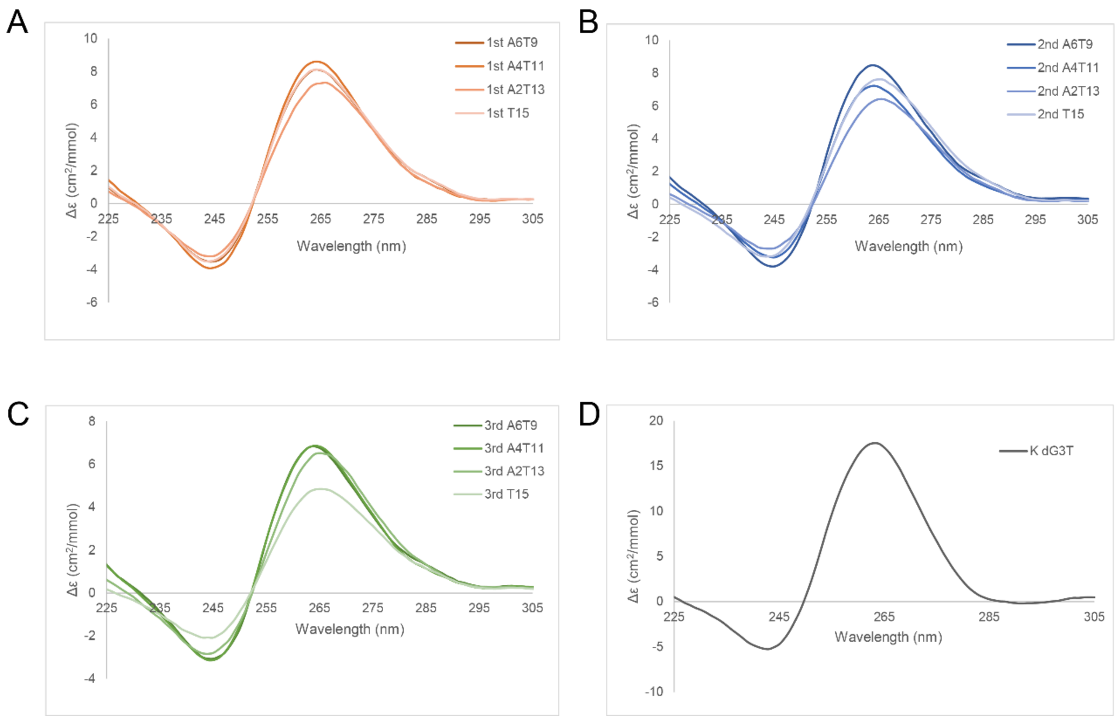

Fig. S2 CD spectra of A6T9, A4T11, A2T13 as well as dG3T in different loop positions in 150 mM K^+. (A), (B) and (C) are the CD spectra of loops with different number of base pairs in first, second, and third loop respectively. (D) is the CD spectra of dG3T.

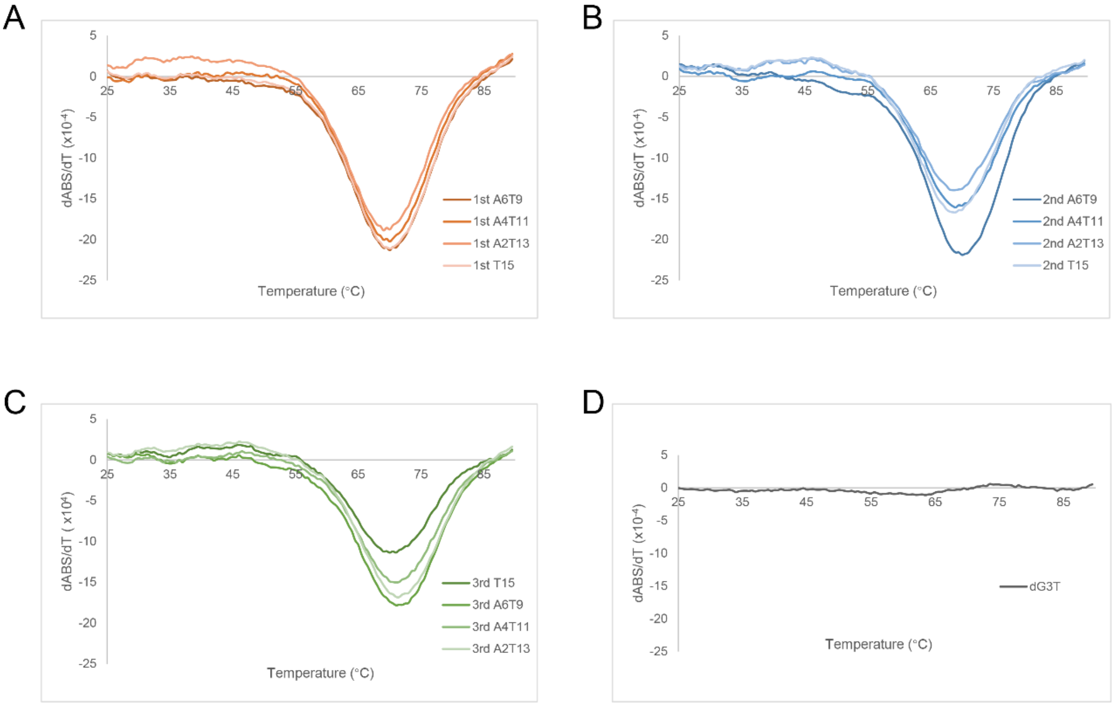

Fig. S3 UV melting spectra of A6T9, A4T11, A2T13 as well as dG3T in different loop positions in 150 mM K^+. (A), (B) and (C) are the UV melting spectra of loops with different number of base pairs in first, second, and third loop respectively. (D) is the UV melting spectra of dG3T. The melting temperature (Tm) is too high (>90°C) under 150 mM K^+.

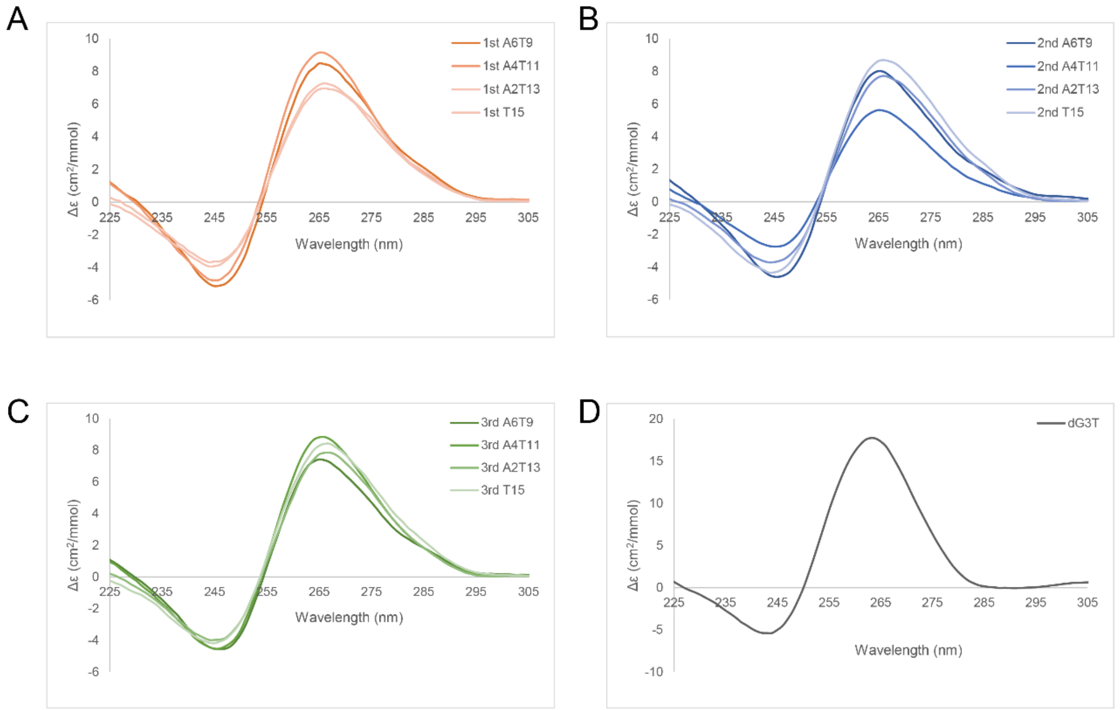

Fig. S4 CD spectra of A6T9, A4T11, A2T13 as well as dG3T in different loop positions in 15 mM K^+. (A), (B) and (C) are the CD spectra of loops with different number of base pairs in first, second, and third loop respectively. (D) is the CD spectra of dG3T.

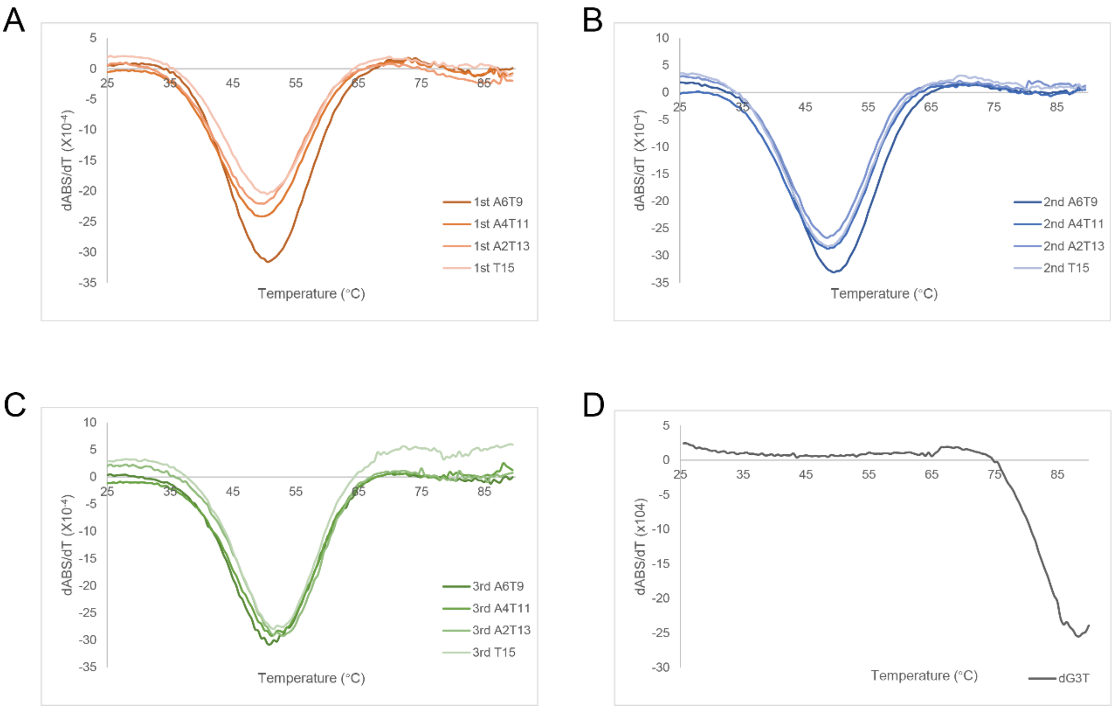

Fig. S5 UV melting spectra of A6T9, A4T11, A2T13, T15 as well as dG3T in different loop positions in 15 mM K^+. (A), (B) and (C) are the UV melting spectra of loops with different number of base pairs in first, second, and third loop respectively. (D) is the UV melting spectra of dG3T.

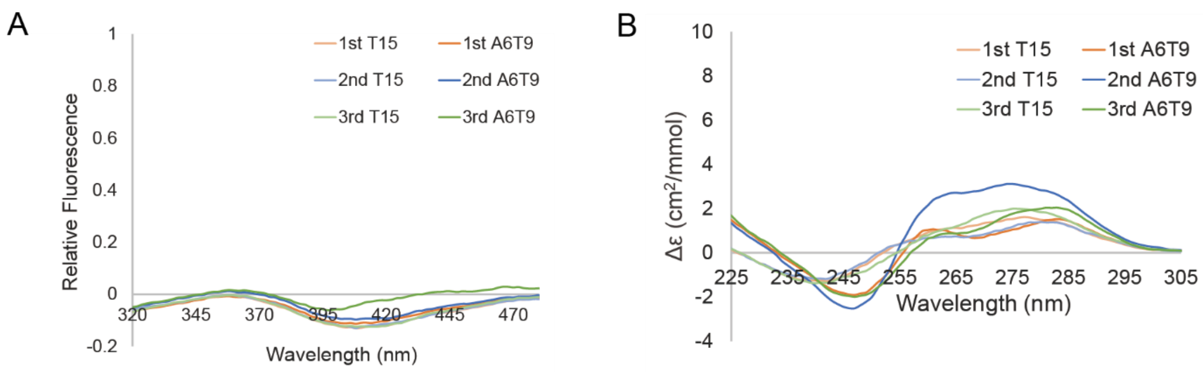

Fig. S6 Relative fluorescence spectra and CD spectra of oligos with T15 and A6T9 loop in different loop positions in 15 mM Li^+. (A) The relative fluorescence; (B) The CD spectra.

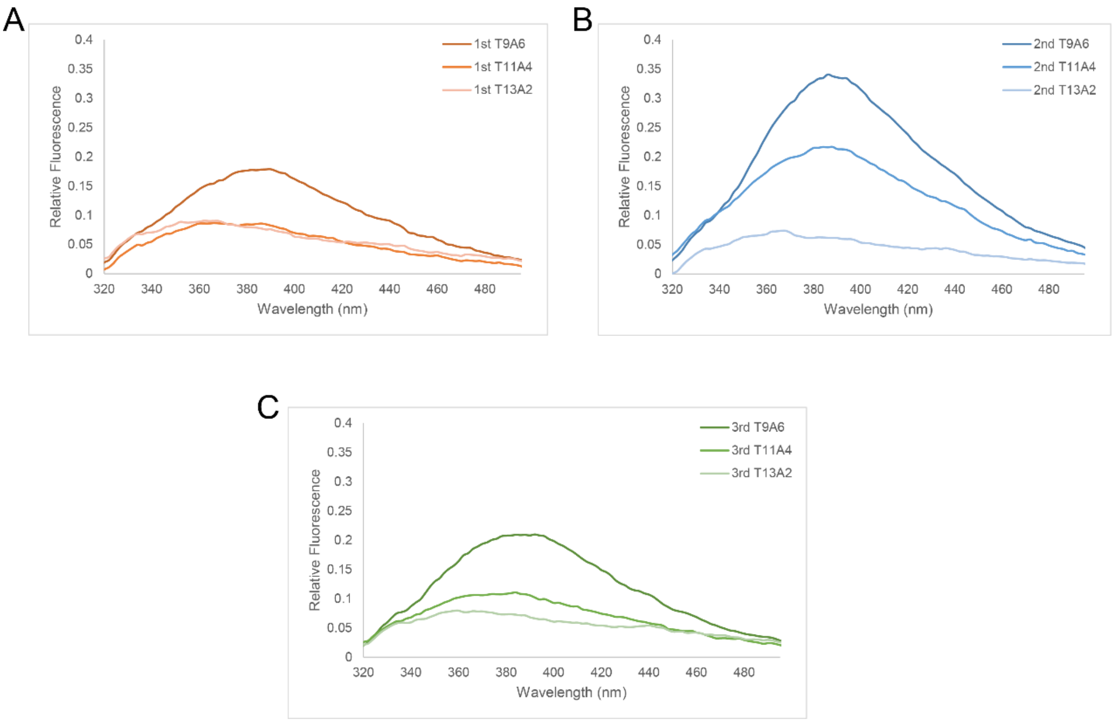

Fig. S7 Relative intrinsic fluorescence plots of T9A6, T11A4 and T13A2 in different loop positions in 15 mM K^{+}. (A), (B) and (C) are the representative plot of T9A6, T11A4 and T13A2 in first, second, and third loop position respectively.

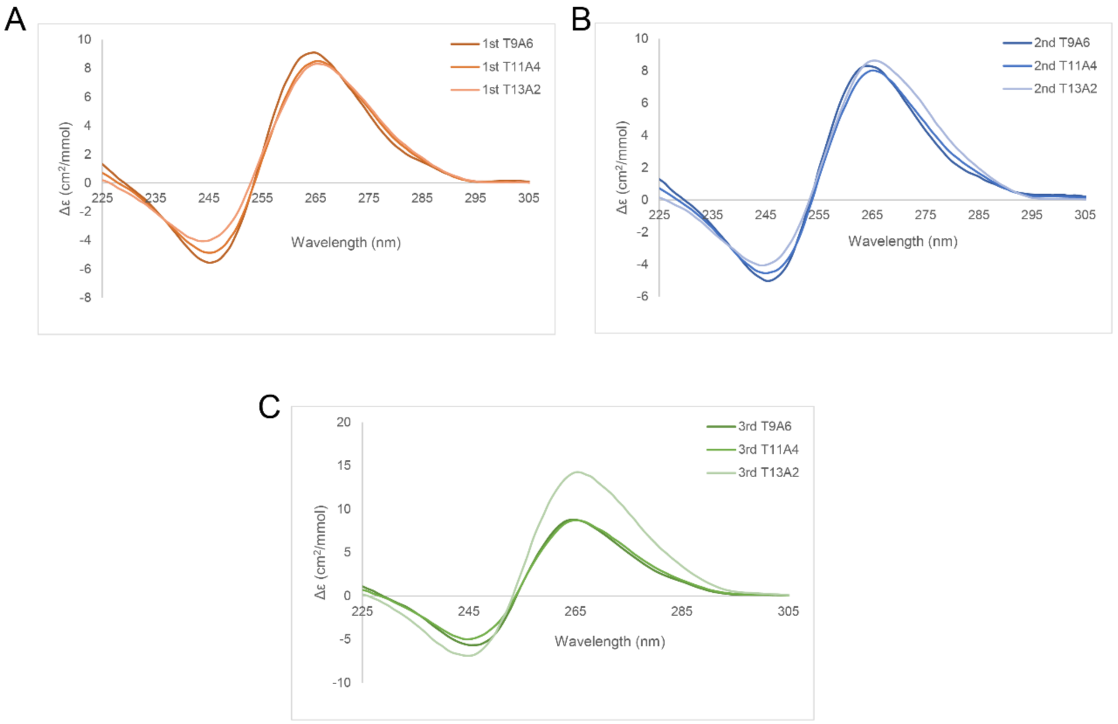

Fig. S8 CD spectra of T9A6, T11A4 and T13A2 in different loop positions in 15 mM K^+. (A), (B) and (C) are the CD spectra of T9A6, T11A4 and T13A2 in first, second, and third loop position respectively.

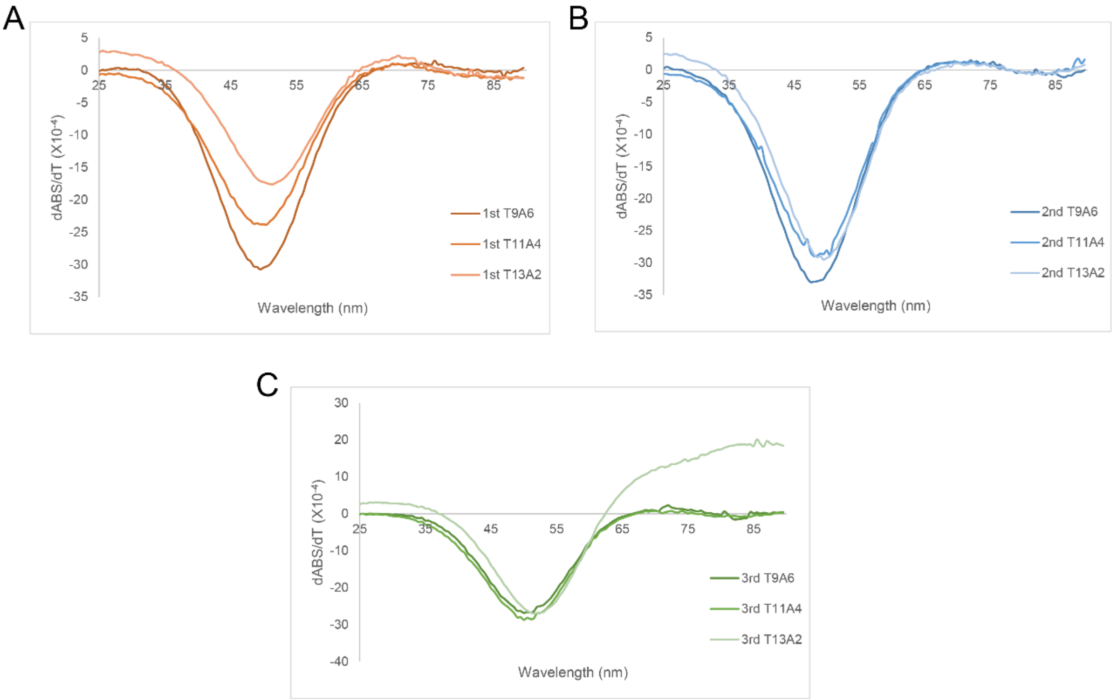

Fig. S9 UV melting spectra of T9A6, T11A4 and T13A2 in different loop positions in 15 mM K^+. (A), (B) and (C) are the CD spectra of T9A6, T11A4 and T13A2 in first, second, and third loop position respectively.

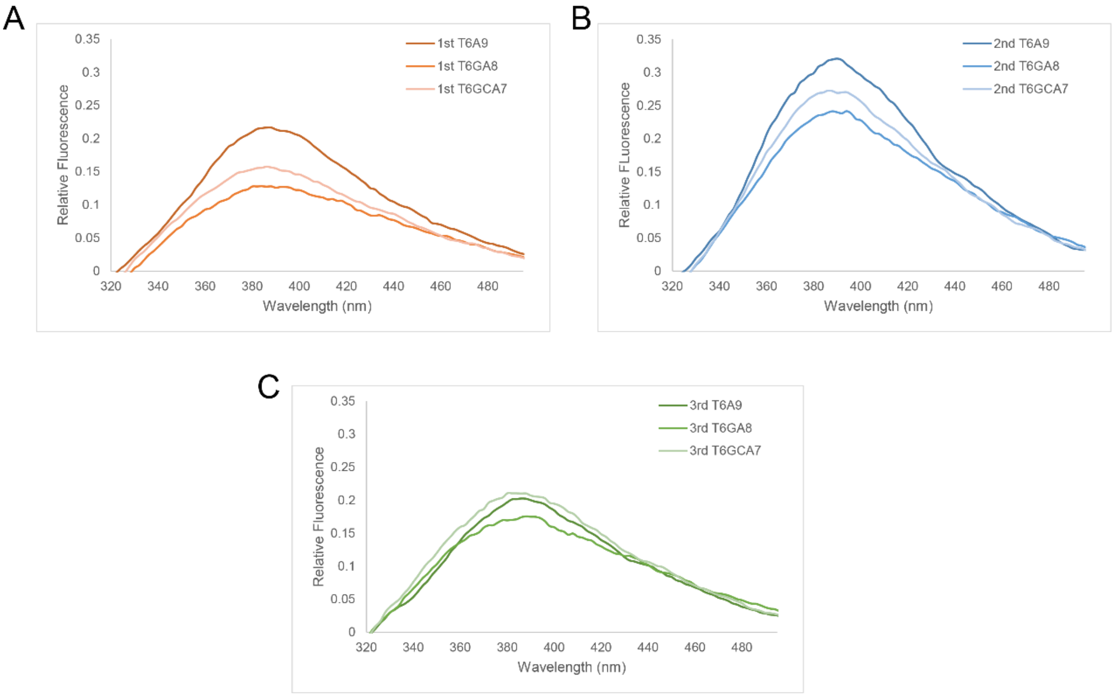

Fig. S10 Relative intrinsic fluorescence plots of T6A9, T6GA8 and T6GCA7 in different loop positions in 15 mM K^+. (A), (B) and (C) are the representative plot of T6A9, T6GA8 and T6GCA7 in first, second, and third loop position respectively.

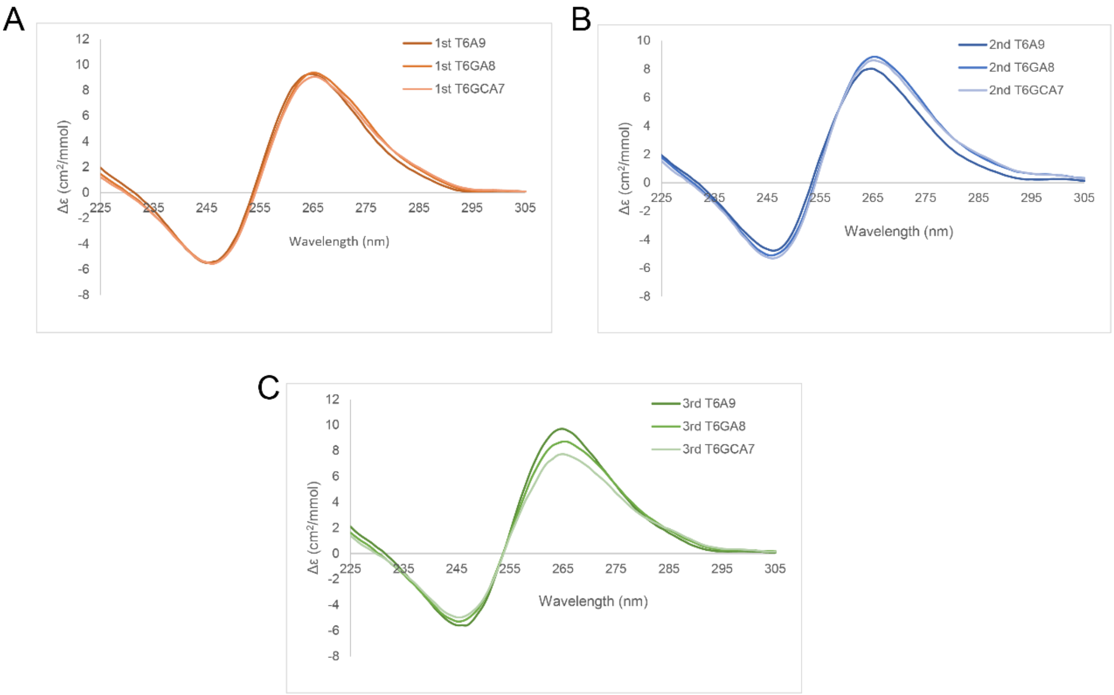

Fig. S11 CD spectra of T6A9, T6GA8 and T6GCA7 in different loop positions in 15 mM K^+. (A), (B) and (C) are the CD spectra of T6A9, T6GA8 and T6GCA7 in first, second, and third loop position respectively.

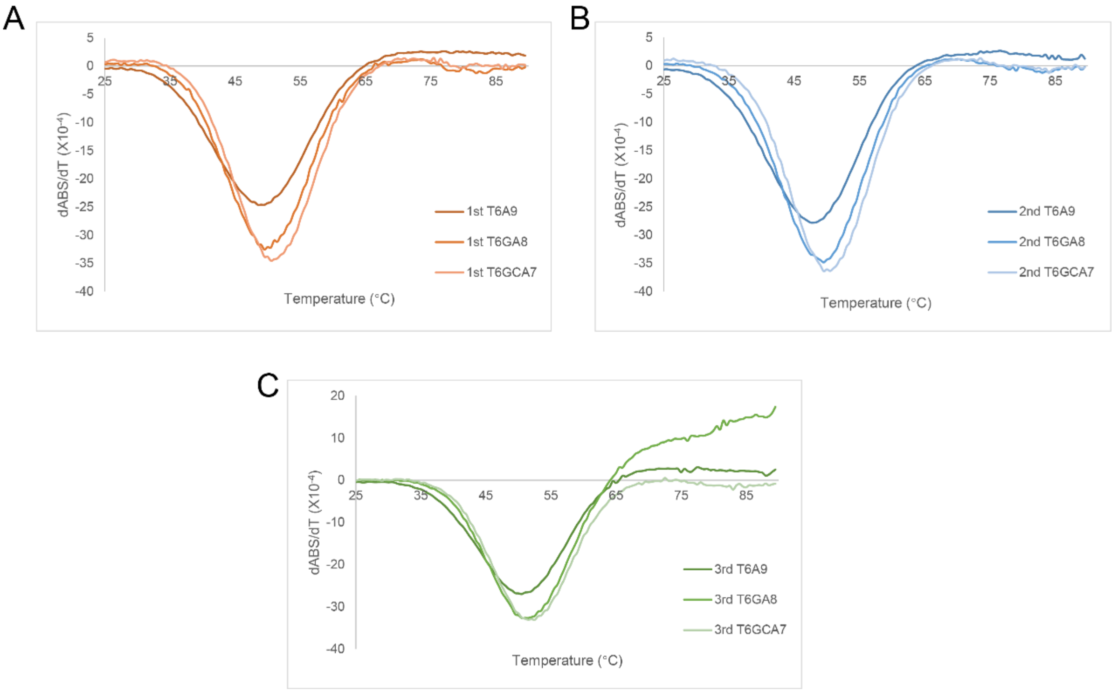

Fig. S12 UV melting spectra of T6A9, T6GA8 and T6GCA7 in different loop positions in 15 mM K^+. (A), (B) and (C) are the CD spectra of T6A9, T6GA8 and T6GCA7 in first, second, and third loop position respectively.

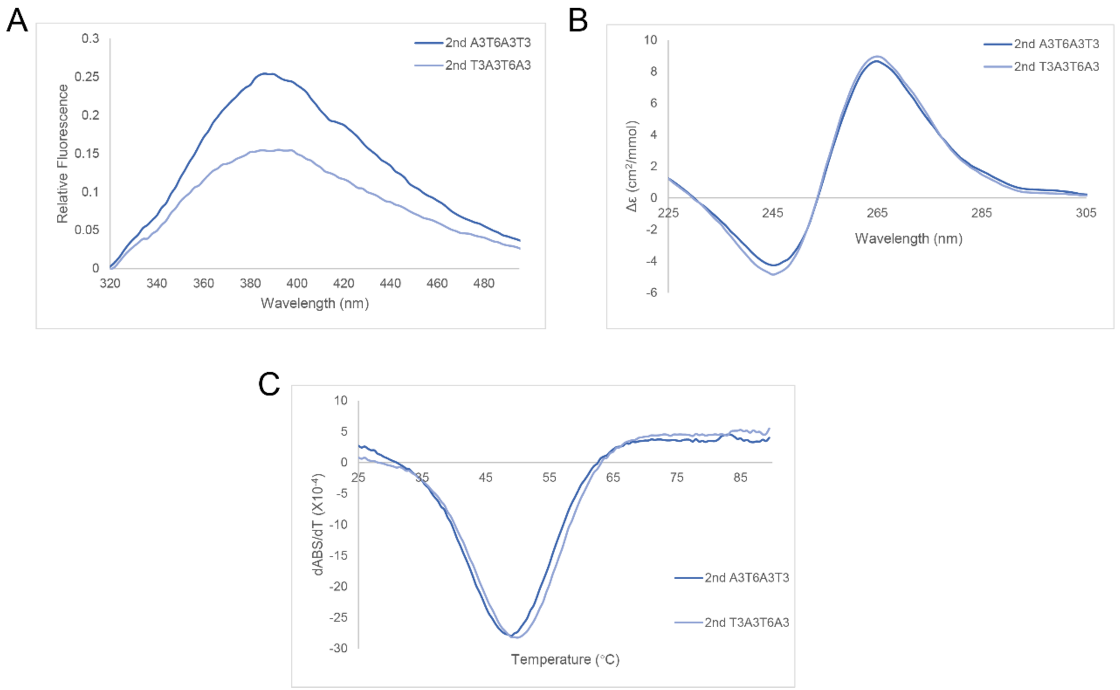

Fig. S13 Relative intrinsic fluorescence plots, CD and UV melting spectra of 2nd A3T6A3T3 and 2nd T3A3T6A3 in 15 mM K^+. (A) Relative intrinsic fluorescence plots, (B) CD spectra, (C) UV melting spectra

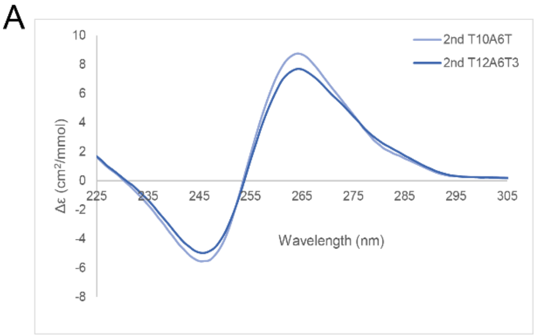

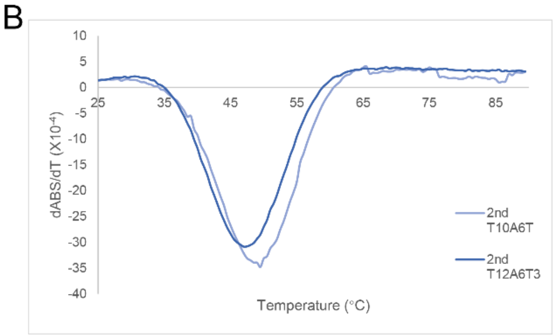

Fig. S14 CD and UV melting spectra of 2nd T10A6T and 2nd T12A6T3 in 15 mM K^+. (A) CD spectra, (B) UV melting spectra.

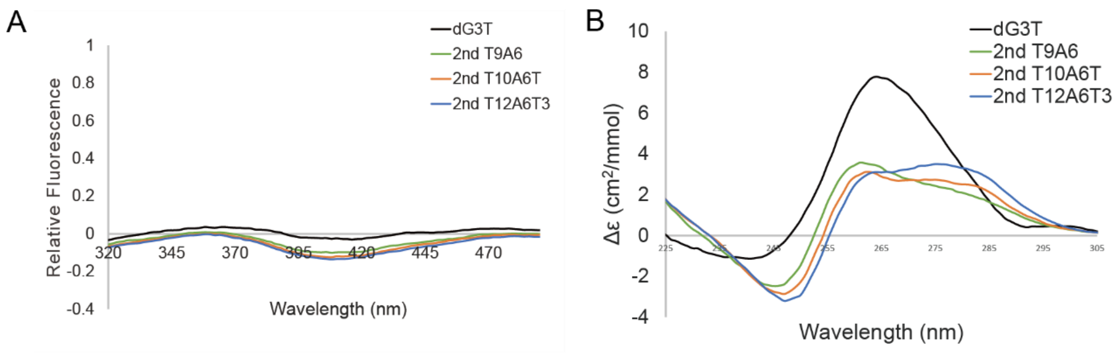

Fig. S15 Intrinsic fluorescence and CD spectra of dG3T, 2nd T9A6, 2nd T10A6T and 2nd T12A6T3 in 15 mM Li^{+}. (A) Intrinsic spectra, (B) CD spectra

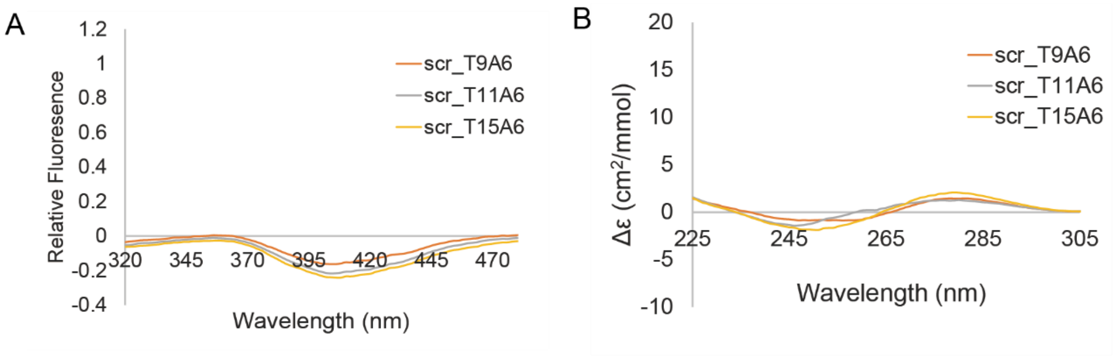

Fig. S16 Intrinsic fluorescence and CD spectra of scrambled oligos in 15 mM K^+. (A) Intrinsic spectra, (B) CD spectra

References

1. C. K. Kwok, M. E. Sherlock and P. C. Bevilacqua, *Biochemistry*, 2013, **52**, 3019-3021.
2. C. K. Kwok, Y. Ding, S. Shahid, S. M. Assmann and P. C. Bevilacqua, *Biochem J*, 2015, **467**, 91-102.
3. T. R. Sosnick, *Curr. Protoc. Nucleic Acid Chem.*, 2001, **Chapter 11**, Unit 11 15.
4. M. E. Sherlock, C. A. Rumble, C. K. Kwok, J. Breffke, M. Maroncelli and P. C. Bevilacqua, *The Journal of Physical Chemistry B*, 2016, **120**, 5146-5158.
5. C. Y. Chan, M. I. Umar and C. K. Kwok, *Chem. Commun. (Camb)*, 2019, **55**, 2616-2619.
